# Editorial: Advancing public health through generative artificial intelligence: a focus on digital well-being and the economy of attention

**DOI:** 10.3389/fpubh.2026.1876366

**Published:** 2026-06-01

**Authors:** Oscar Robayo-Pinzon, Sandra Rojas-Berrio, Jorge E. Camargo, Gordon R. Foxall

**Affiliations:** 1 School of Management and Business, Universidad del Rosario, Bogotá, Colombia; 2 Faculty of Economics, Universidad Nacional de Colombia, Bogotá, Colombia; 3 Faculty of Engineering, Universidad Nacional de Colombia, Bogotá, Colombia; 4 Cardiff Business School, Cardiff University, Cardiff, United Kingdom; 5 School of Management, Reykjavik University, Reykjavik, Iceland

**Keywords:** conversational agents, digital wellbeing, disease surveillance, economy of attention, generative artificial intelligence (GenAI), mental health, personalized healthcare, public health

The rapid evolution of generative artificial intelligence (GenAI) is reshaping the foundations of public health, not only by enhancing analytical capabilities and service delivery, but also by transforming how individuals engage with information, healthcare systems, and their own wellbeing. The contributions in this Research Topic collectively demonstrate that AI is no longer a peripheral tool; it is becoming an infrastructural component of contemporary public health. At the same time, they reveal a critical tension: while AI expands opportunities for personalization, efficiency, and prediction, it also introduces new behavioral, ethical, and systemic challenges that must be addressed to ensure equitable and sustainable health outcomes.

A first set of contributions highlights the role of AI in strengthening public health systems and resilience. Empirical evidence shows that AI technologies can significantly enhance urban public health resilience by improving resource allocation, response efficiency, and cross-regional collaboration, although their benefits remain unevenly distributed due to disparities in technological capacity (Chen and Zhang). Complementing this systems-level perspective, the systematic review of AI-driven early warning systems demonstrates how machine learning and natural language processing enable earlier detection of infectious disease outbreaks through the integration of heterogeneous data sources (Villanueva-Miranda, Xiao et al.). Together, these studies underline AI's potential to shift public health from reactive to anticipatory models. However, they also converge on persistent limitations—data quality, infrastructure gaps, and the need for interdisciplinary coordination—pointing to the importance of governance frameworks that align technological innovation with public health priorities.

A second cluster of articles focuses on AI-enabled personalization in healthcare delivery, illustrating how generative and predictive models are redefining patient-centered care. The development of retrieval-augmented generation systems for physician recommendation demonstrates how large language models can improve triage accuracy and scalability in web-based medical services (Zheng et al.). Similarly, deep learning architectures applied to diagnostic recommendation systems show measurable improvements in clinical decision support, highlighting the capacity of AI to integrate complex medical knowledge into actionable insights (Wang et al.). From the user perspective, qualitative evidence on mHealth applications emphasizes the importance of personalization, usability, and privacy in shaping engagement, particularly among populations such as college students facing growing mental health challenges (Hoglund et al.). Collectively, these studies suggest that personalization is not merely a technical feature but a central determinant of effectiveness in digital health interventions. Yet they also raise critical questions about transparency, reliability, and the risk of over-reliance on automated systems in clinical contexts.

The third thematic line addresses the intersection of digital wellbeing, mental health, and the economy of attention, a domain where generative AI exerts both therapeutic and disruptive influences. Research on virtual humans indicates their potential to support mood management and psychological wellbeing, particularly among individuals who prefer online social interaction, although acceptance depends on perceived usefulness and ease of use (Lee et al.). At the same time, behavioral evidence suggests that while current levels of perceived dependence on GenAI tools remain relatively low, patterns of reinforcement and immediate gratification may increase the relative value of AI-mediated interactions, raising concerns about long-term dependence and its implications for mental health (Robayo-Pinzon et al.). These findings are complemented by systematic analyses showing how sentiment analysis and large language models can extract population-level insights from digital communication, enabling more responsive public health interventions while also introducing challenges related to bias, representativeness, and ethics (Villanueva-Miranda, Xie et al.). Finally, emerging perspectives on the clinical integration of conversational agents emphasize the need for personalization, multimodality, and ethical safeguards to ensure that AI systems augment rather than replace human care (Frisone et al.).

Taken together, the articles in this Research Topic point to a fundamental reconfiguration of public health across three interconnected dimensions: prediction, personalization, and participation. AI enhances prediction through data-driven surveillance and early warning systems; it enables personalization through adaptive, context-aware interventions; and it reshapes participation by mediating how individuals interact with health information, services, and social environments. However, this transformation also introduces a fourth, less visible dimension: attention. As AI systems compete for and shape user attention, they influence behavioral patterns, decision-making processes, and ultimately health outcomes. The emerging “economy of attention” thus becomes a critical public health concern, requiring new conceptual and methodological approaches.

Considering these developments, this Research Topic suggests several priorities for future research and policy. First, there is a need to address structural inequalities in AI deployment, ensuring that technological benefits are distributed equitably across regions and populations. Second, advancing model transparency and explainability is essential to build trust and support informed decision-making among both clinicians and patients. Third, research should further investigate the behavioral and psychological impacts of sustained interaction with AI systems, particularly in relation to dependence, autonomy, and wellbeing. Fourth, the development of robust ethical and regulatory frameworks must accompany technological innovation, with particular attention to privacy, bias, and accountability. Finally, interdisciplinary collaboration between public health experts, data scientists, behavioral economists, and policymakers will be crucial to align AI development with societal health goals.

Ultimately, the contributions in this issue move beyond a narrow focus on technological performance to highlight a broader challenge: how to design AI systems that not only optimize efficiency and accuracy, but also support human flourishing in increasingly digital environments. Public health in the age of generative AI will depend not only on what these systems can do, but on how they are integrated into the social, behavioral, and institutional construction of everyday life.

